# Disparities in initiating standard first-line cancer treatment and survival for patients with an immigrant background: a Danish register-based cohort study

**DOI:** 10.2340/1651-226X.2026.46256

**Published:** 2026-09-18

**Authors:** Emma N. Ishøy, Maria Kristiansen, Ismail Gögenur, Erik Jakobsen, Lars Lund, Fie Stegenborg, Susanne Rosthøj, Mia K. Grand, Pernille Bidstrup, Morten Sodemann, Susanne O. Dalton

**Affiliations:** a Cancer Survivorship, Danish Cancer Institute, Copenhagen, Denmark; b Danish Research Center for Equality in Cancer, Zealand University Hospital, Næstved, Denmark; c Department of Public Health, Faculty of Health and Medical Sciences, University of Copenhagen, Copenhagen, Denmark; d Center for Surgical Science, Department of Surgery, Zealand University Hospital, Køge, Denmark; e Department of Cardiac, Thoracic and Vascular Surgery, Odense University Hospital, Odense, Denmark; f Department of Urology, Odense University Hospital, Odense, Denmark; g Statistics and Data Analysis, Danish Cancer Institute, Copenhagen, Denmark; h Psychological Aspects of Cancer, Cancer Survivorship, Danish Cancer Institute, Copenhagen, Denmark; i Department of Infectious Diseases, Odense University Hospital, Odense, Denmark; j Institute of Clinical Medicine, Faculty of Health Sciences, University of Copenhagen, Copenhagen, Denmark

**Keywords:** Immigrants and emigrants, ethnic and racial minorities, neoplasms, socioeconomic disparities in health, survival, mortality

## Abstract

**Background and purpose:**

Individuals with an immigrant background may face barriers to healthcare. However, evidence on differences in cancer treatment and survival between patients with an immigrant background and patients of Danish origin is limited.

**Patients and methods:**

We included patients aged ≥50 years diagnosed with a first primary colon, lung, prostate, or kidney cancer between 2011 and 2022 from national clinical cancer databases. First-line treatment initiation (30/90 days) and survival (1/5 years) were assessed using risk ratios (RRs) comparing patients with an immigrant background to those of Danish origin. Immigrant backgrounds were categorized according to their origin in (1) other Western countries, (2) Central and Eastern Europe, and (3) the Middle East, North Africa, and Central Asia.

**Results:**

Overall, there were no systematic differences in the initiation of first-line treatment or survival between patients with an immigrant background and patients of Danish origin. Exceptions included lower probability of initiating treatment (30-day RR: 0.76 [95% confidence interval (CI): 0.62; 0.93] and 90-day RR: 0.92 [95% CI: 0.87; 0.98]) and 1-year risk of death (RR: 0.85 [95% CI: 0.77; 0.93]) among lung cancer patients from Central and Eastern Europe, lower 30-day treatment initiation among patients from the Middle East, North Africa, and Central Asia (RR: 0.80 [95% CI: 0.64; 1.00]), and higher 5-year risk of death among prostate cancer patients from other Western countries (RR: 1.16 [95% CI: 1.01; 1.34]).

**Interpretation:**

Overall, patients with an immigrant background had similar initiation of first-line cancer treatment and survival to those of Danish origin, with some statistically significant differences but no consistent pattern across cancer types or regions of origin.

## Introduction

Over the past decades, international migration has increased substantially, resulting in growing immigrant populations worldwide [1]. In 2024, immigrants comprised 12% of the Danish population, and this proportion is expected to continue growing [2]. Previous studies suggest that healthcare utilization patterns among immigrants differ noticeably from those of the native-born population [3, 4]. In Denmark, most healthcare services are financed by general taxes, including cancer treatment, screening, and vaccination programs. Although the Danish healthcare system is based on the principles of free and equal access to healthcare for all citizens, disparities in healthcare utilization persist among immigrant groups [5–7]. These disparities in healthcare utilization are multifactorial, encompassing patient-level barriers such as lower socioeconomic status, lack of social support, limited local language skills, and lack of awareness of service availability [4]. Both provider- and system-level factors, such as limited cultural competence and organizational factors, further exacerbate inequalities [4]. Together, these barriers raise concerns regarding timely access to appropriate cancer care for individuals with an immigrant background.

In 2008, Denmark implemented standardized cancer patient pathways based on evidence-based guidelines to improve timely diagnosis and high-quality treatment nationwide. Additionally, treatment is individualized based on patient and disease characteristics, as well as patient preferences [8]. Several Danish register-based studies have documented lower participation in cancer prevention measures, including screening programs and HPV vaccination [9–12] and differences in cancer stage at diagnosis among immigrants [13]. However, emerging evidence finds that cancer survival does not differ between immigrants and Danish-born individuals [14–17].

International research, predominantly from the United States, where healthcare access differs substantially from universal and hybrid healthcare systems predominant in Europe, has consistently highlighted disparities in cancer treatment based on race or ethnicity [18–21]. However, evidence from Europe on ethnicity and cancer care remains limited [22], and immigrant populations may face additional migration-related barriers, including language barriers and unfamiliarity with the healthcare system. As immigrant populations continue to grow and age [1, 23], an increasing number of individuals with an immigrant background will need cancer care, highlighting the importance of understanding potential disparities in cancer care and outcomes. While one Danish study found no differences in cancer treatment among immigrants diagnosed with acute myeloid leukemia [15], no research has focused on solid tumors, and no research has examined differences by specific regions of origin. Thus, there is a need for studies investigating access to first-line cancer treatment across multiple cancer sites within a universal healthcare system. The present study addresses this gap by examining differences in initiation of first-line cancer treatment within 30 and 90 days after diagnosis among patients with immigrant background compared with patients of Danish origin across lung, colon, prostate, and kidney cancer.

## Method

### Data sources

We used data from the national clinical databases comprising the Danish Colorectal Cancer Group database (DCCG) [24], Danish Lung Cancer Group (DLCG) [25], Danish Prostate Cancer Group (DaProCa) [26], and Danish Renal Cancer Group (DaRenCa) [27]. From these databases, we obtained information on clinical variables. Patients identified in these databases were linked with data from Danish nationwide registers to obtain information on socio-demographics, health, and migration-related variables [28].

### Region of origin

This study includes individuals of Danish origin and individuals with immigrant background. In accordance with Statistics Denmark’s definition, immigrants were defined as individuals born abroad, with no parent who was both a Danish citizen and born in Denmark. The country of origin of the individuals was based on the mother’s country of birth if known; otherwise, the country of birth of the father was used. If parental information was unavailable, the country of origin was based on the individual’s own information on the country of birth [29]. Information on origin, including immigrant background and country of origin, was obtained from the Danish Civil Registration System [30]. We categorized regions inspired by Jamison et al. [31], but combined regions due to few observations. This resulted in the categories: (1) Denmark, (2) other Western countries (North Atlantic, the United States, Australia, and New Zealand), (3) Central and Eastern Europe, and (4) the Middle East, North Africa, and Central Asia. Other geographical areas, including Southeast Asia and Pacific Western, Sub-Saharan Africa, and Latin America and the Caribbean, were not included due to few observations limiting meaningful categories. Included countries are listed in the Supplementary material (Table S1).

### Study population

**Figure 1:**
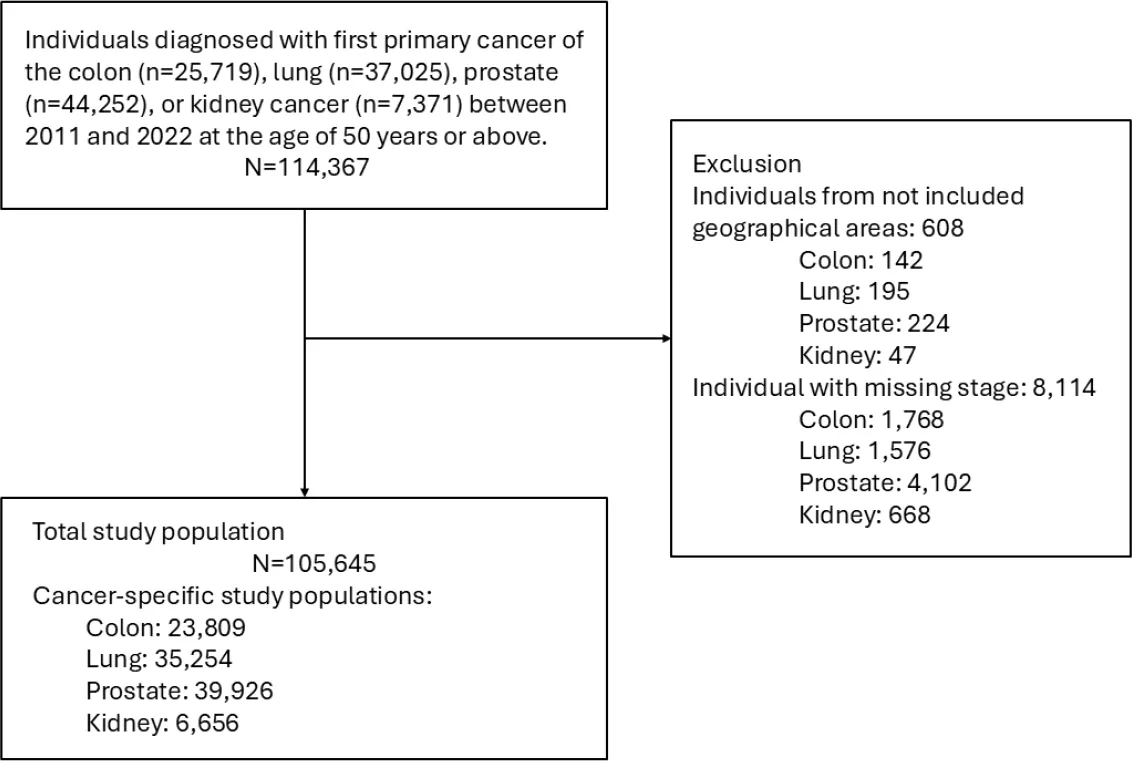
Flow chart of the study population of patients aged ≥50 years diagnosed with colon, lung, prostate, or kidney cancer in Denmark between 2011 and 2022.

All patients registered with a first primary cancer of the colon, lung, prostate, or kidney in the respective clinical databases between 2011 and 2022, aged over 50 years, were included. For patients with lung or colon cancer, only those diagnosed with non-small cell lung cancer and those with non-acute surgical colon cancer, respectively, were included. Patients with missing information on stage at diagnosis and patients from the not included geographical areas were excluded from the study population (Figure 1).

### First-line cancer treatment

Matrices of standard first-line cancer treatment according to national Danish guidelines, based on stage at diagnosis, were developed in collaboration with specialized cancer clinicians (Tables S2–5). We obtained information on stage at diagnosis and treatment (and corresponding dates for treatment) from the national clinical databases, and additional information on treatment was obtained from the Danish National Patient Registry. We categorized the patients according to whether they initiated standard first-line cancer treatment (no; yes). First-line treatment initiation was evaluated at 30 and 90 days after diagnosis. The 30-day time point was chosen to reflect early treatment initiation and potential differences in timely access to first-line treatment, whereas the 90-day time point reflects treatment initiation within a clinically acceptable time frame. Throughout the manuscript, “first-line cancer treatment” is used as an umbrella term for guideline-recommended standard first-line management, including active surveillance and watchful waiting.

### All-cause mortality

Information on the date of death from all causes was obtained from the Danish Civil Registration System until emigration or the 31st of December 2022 [30].

### Patient characteristics

Information on the patients’ sex, age at diagnosis, education, cohabitation status, and health (Charlson Comorbidity Index [CCI] and psychiatric disorders) was obtained from the Danish registers at the time of diagnosis [28]. Information on cohabitation status was defined as cohabiting with a partner or not. Information on the highest attained education was categorized as short (corresponding to ISCED level 1–2), medium (ISCED level 3–4), and longS2 education (ISCED level 5–8). We used CCI, including diagnoses 5 years prior to the cancer diagnosis (none or any). Psychiatric disorders (psychotic diseases, depression, anxiety, and other neurotic, stress-related, and somatoform disorders) were included 5 years prior to cancer diagnosis (none or any). Calendar period of diagnosis (prior to or after 2016) was included as the Danish national cancer care packages were updated in 2016. For patients diagnosed with colon and lung cancer, information on Eastern Cooperative Oncology Group (ECOG) performance status was available from the clinical databases. For patients diagnosed with colon cancer, we included information on: deficient mismatch repair (dMMR), signet ring cell carcinoma, and T4, all of which are tumor characteristics associated with prognosis. For patients with an immigrant background, we obtained information on the number of years since their first immigration to Denmark.

### Statistical analyses

We calculated frequencies and proportions for categorical baseline characteristics and medians for continuous variables across the four cancer sites by regions of origin. Additionally, we identified the 10 most common countries of origin among patients with an immigrant background.

Patients were followed from the date of cancer diagnosis until initiation of first-line treatment, death from any cause, or end of follow-up, whichever occurred first. Initiation of treatments other than the guideline-recommended first-line treatment did not result in end of follow-up. All analyses were performed separately for each cancer site. Cumulative incidence curves of first-line treatment initiation were estimated using the Aalen–Johansen estimator, accounting for death as a competing risk. The curves were graphically illustrated up to 90 days of follow-up, and absolute risks (cumulative incidence proportions) were derived at 30 and 90 days. Estimation of risk ratios (RRs) of the 30- and 90-day cumulative incidences of initiating first-line treatment was performed using an approach based on pseudo-observations and generalized estimating equations (GEE) with a log-link and the independence covariance working matrix [32]. RRs were estimated using two adjusted models: (1) adjusted for sex, age at diagnosis, and year of diagnosis, and (2) additionally adjusted for cohabitation status, education, CCI, psychiatric disorders, stage, and cancer-specific variables (Colon cancer: ECOG performance status, dMMR, signet ring cell carcinoma, and T4. Lung cancer: ECOG performance status) to examine how the estimated associations changed after additional adjustment for sociodemographic and clinical characteristics. These variables were included because they are important prognostic factors. The same approach was applied to estimate absolute risks and RRs of death at 1 and 5 years after diagnosis. Median follow-up time, censored at 5 years after diagnosis, was estimated by the reverse Kaplan-Meier estimator [33].

Missing values in education and the cancer-specific variables were handled using multiple imputation by chained equations under a missing at random assumption [34]. The imputation model included immigrant background, all variables included in the analyses, and the corresponding pseudo-observations. Estimates of RRs and standard errors were pooled using Rubin’s rules based on 10 imputed datasets.

We performed analyses of first-line treatment and death stratified by stage (local/regional and advanced stage [definition Table S6]). We performed the first-line treatment initiation analyses with the immigrant population categorized according to years lived in Denmark.

All analyses were performed using the statistical software R version 4.3.2 with the package eventglm for the main analysis. Estimates are presented with 95% confidence intervals. Given the large number of statistical tests, we emphasize overall patterns rather than isolated statistically significant findings.

## Results

### Patient characteristics

A total of 105,645 patients were included. Of them, 101,320 (96%) of the patients were of Danish origin, while 2,229 (2%) were from other Western countries, 947 (1%) from Central and Eastern Europe, and 1,149 (1%) from the Middle East, North Africa, and Central Asia. Patients from Central and Eastern Europe and the Middle East, North Africa, and Central Asia were younger at diagnosis (median age of 61–67 years vs. 67–73 years) and more had an ECOG performance status of 0 (colon: 55–58% vs. 62–66% and lung: 39–41% vs.45–50%) compared with patients of Danish origin and patients from other Western countries (Table 1). Compared with patients of Danish origin, patients from the Middle East, North Africa, and Central Asia were more often men, had shorter education, and more often had a CCI score of 0. Patients from the Middle East, North Africa, and Central Asia also had a smaller proportion of colon, prostate, and kidney cancer diagnosed at the most advanced stage compared with all other groups. A smaller proportion of patients from Central and Eastern Europe had lived in Denmark for more than 20 years (60–74% vs. 78–92%), compared with patients from other immigrant backgrounds. The most common countries of origin across cancer sites were Germany, Turkey, Norway, Sweden, and Bosnia and Herzegovina (Table S7).

**Table 1a:** Characteristics of the study population of patients diagnosed in Denmark between 2011 and 2022 with colon, lung, prostate, and kidney cancer, by region of origin.

Characteristics	Colon cancer patients (*N* = 23,809)				Lung cancer patients (*N* = 35,254)			
	Denmark (*n* = 22,859)	Other Western countries (*n* = 505)	Central and Eastern Europe (*n* = 200)	Middle East, North Africa, and Central Asia (*n* = 245)	Denmark (*n* = 33,727)	Other Western countries (*n* = 767)	Central and Eastern Europe (*n* = 411)	Middle East, North Africa, and Central Asia (*n* = 349)
	*n* (%)	*n* (%)	*n* (%)	*n* (%)	*n* (%)	*n* (%)	*n* (%)	*n* (%)
Sex								
Female	11,113 (48)	257 (51)	96 (48)	82 (33)	17,054 (51)	373 (49)	167 (41)	57 (16)
Male	11,746 (51)	248 (49)	104 (52)	163 (67)	16,673 (49)	394 (51)	244 (59)	292 (84)
Age at diagnosis (median [IQR])	72 (65–79)	73 (67–79)	66 (60–74)	65 (58–72)	71 (64–77)	70 (64–75)	66 (60–72)	65 (58–73)
Year of diagnosis								
Before 2016	9,057 (40)	196 (39)	58 (29)	62 (25)	13,072 (39)	292 (38)	128 (31)	108 (31)
2016 or after	13,802 (60)	309 (61)	142 (71)	183 (75)	20,655 (61)	475 (62)	283 (69)	241 (69)
Education*								
Short	6,241 (28)	55 (12)	23 (14)	80 (38)	11,029 (33)	105 (15)	62 (18)	133 (43)
Medium	11,401 (51)	253 (55)	93 (55)	80 (38)	17,294 (52)	434 (62)	209 (59)	123 (40)
Long	4,816 (21)	154 (33)	53 (31)	53 (25)	4,746 (14)	163 (23)	82 (23)	51 (17)
Missing	401 (2)	43 (9)	31 (16)	32 (13)	658 (2)	65 (8)	58 (14)	42 (12)
Partner								
Not cohabiting	8,804 (39)	217 (43)	83 (42)	81 (33)	14,787 (44)	340 (44)	182 (44)	146 (42)
Cohabiting	14,055 (61)	288 (57)	117 (59)	164 (67)	18,940 (56)	427 (56)	229 (56)	203 (58)
Charlson Comorbidity Index								
0	8,714 (38)	202 (40)	81 (41)	127 (52)	17,199 (51)	372 (49)	179 (44)	190 (54)
1+	14,145 (62)	303 (60)	119 (60)	118 (48)	16,528 (49)	395 (51)	232 (56)	159 (46)
Psychiatric disorders								
No	19,795 (87)	443 (88)	170 (85)	203 (83)	26,931 (80)	641 (84)	309 (75)	281 (81)
Yes	3,064 (13)	62 (12)	30 (15)	42 (17)	6,796 (20)	126 (16)	102 (25)	68 (19)
Years in Denmark								
Median (IQR)	-	37 (31–42)	25 (17–35)	31 (26–36)	-	34 (28–40)	25 (18–33)	31 (26–35)
> 20 years	-	462 (91)	125 (63)	206 (84)	-	660 (86)	274 (67)	301 (86)
≤ 20 years	-	43 (9)	75 (38)	39 (16)	-	107 (14)	137 (33)	48 (14)
Stage at diagnosis**								
I	5,531 (24)	129 (26)	47 (24)	69 (28)	7,249 (21)	165 (22)	76 (18)	77 (22)
II	5,406 (24)	113 (22)	40 (20)	50 (20)	3,056 (9)	79 (10)	38 (9)	21 (6)
III	6,528 (29)	143 (28)	62 (31)	81 (33)	6,817 (20)	147 (19)	103 (25)	73 (21)
IV	5,394 (24)	120 (24)	51 (26)	45 (18)	16,605 (49)	376 (49)	194 (47)	178 (51)
ECOG performance status*								
0 score	9,958 (58)	207 (55)	96 (62)	135 (66)	12,466 (39)	296 (41)	194 (50)	150 (45)
1–4 score***	7,092 (42)	167 (45)	60 (38)	69 (34)	19,133 (61)	418 (59)	196 (50)	187 (55)
Missing	5,809 (25)	131 (26)	44 (22)	41 (17)	2,128 (6)	53 (7)	21 (5)	12 (3)

* Percentages are based on non-missing observations. Missing is reported as the percentage of the total group.

** Prostate cancer is based on D’Amico score, going 1, 2, 3, -1.

*** Score 5 for patients diagnosed with lung cancer is included.

**Table 1b:** Characteristics of the study population of patients diagnosed in Denmark between 2011 and 2022 with colon, lung, prostate, and kidney cancer, by region of origin.

Characteristics	Prostate cancer patients (*N* = 39,926)				Kidney cancer patients (*N* = 6,656)			
	Denmark (*n* = 38,440)	Other Western countries (*n* = 815)	Central and Eastern Europe (*n* = 253)	Middle East, North Africa, and Central Asia (*n* = 418)	Denmark (*n* = 6,294)	Other Western countries (*n* = 142)	Central and Eastern Europe (*n* = 83)	Middle East, North Africa, and Central Asia (*n* = 137)
	*n* (%)	*n* (%)	*n* (%)	*n* (%)	*n* (%)	*n* (%)	*n* (%)	*n* (%)
Sex								
Female	-	-	-	-	2,003 (32)	38 (27)	39 (47)	31 (23)
Male	38,440 (100)	815 (100)	253 (100)	418 (100)	4,291 (68)	104 (73)	44 (53)	106 (77)
Age at diagnosis (median [IQR])	70 (65–76)	70 (64–75)	67 (60–72)	66 (59–72)	67 (60–74)	68 (59–74)	61 (56–70)	61 (55–69)
Year of diagnosis								
Before 2016	14,754 (38)	308 (38)	82 (32)	117 (28)	2,242 (36)	40 (28)	25 (30)	39 (28)
2016 or after	23,686 (62)	507 (62)	171 (68)	301 (72)	4,052 (64)	102 (72)	58 (70)	98 (72)
Education*								
Short	7,571 (20)	30 (4)	29 (13)	109 (28)	1,528 (25)	14 (11)	16 (22)	41 (34)
Medium	20,166 (53)	384 (52)	122 (54)	165 (42)	3,372 (54)	67 (51)	41 (55)	51 (43)
Long	10,114 (27)	323 (44)	77 (34)	116 (30)	1,302 (21)	51 (39)	17 (23)	28 (12)
Missing	589 (2)	78 (10)	25 (10)	28 (7)	92 (1)	10 (7)	9 (11)	17 (12)
Partner								
Not cohabiting	9,386 (24)	228 (28)	63 (25)	124 (30)	2,101 (33)	46 (32)	36 (43)	36 (26)
Cohabiting	29,054 (76)	587 (72)	190 (75)	294 (70)	4,193 (67)	96 (68)	47 (57)	101 (74)
Charlson Comorbidity Index								
0	12,322 (32)	283 (35)	85 (34)	188 (45)	2,737 (43)	59 (42)	30 (36)	73 (53)
1+	26,118 (68)	532 (65)	168 (66)	230 (55)	3,557 (57)	83 (58)	53 (64)	64 (47)
Psychiatric disorders								
No	34,625 (90)	741 (91)	220 (87)	342 (82)	5,382 (86)	125 (88)	67 (81)	113 (82)
Yes	3,815 (10)	74 (9)	33 (13)	76 (18)	912 (14)	17 (12)	16 (19)	24 (18)
Years in Denmark								
Median (IQR)	-	34 (28–39)	27 (20–36)	32 (27–36)	-	33 (27–39)	23 (18–30)	30 (22–34)
> 20 years	-	691 (85)	186 (74)	356 (85)	-	124 (87)	50 (60)	107 (78)
≤ 20 years	-	124 (15)	67 (26)	62 (15)	-	18 (13)	33 (40)	30 (22)
Stage at diagnosis**								
I	4,608 (12)	117 (14)	41 (16)	60 (14)	3,292 (52)	82 (58)	46 (55)	91 (66)
II	12,458 (32)	264 (32)	83 (33)	168 (40)	539 (9)	11 (8)	5 (6)	9 (7)
III	15,752 (41)	341 (42)	105 (42)	161 (39)	1,305 (21)	26 (18)	15 (18)	19 (14)
IV	5,622 (15)	93 (11)	24 (9)	29 (7)	1,158 (18)	23 (16)	17 (20)	18 (13)
ECOG performance status*								
0 score	-	-	-	-	-	-	-	-
1–4 score***	-	-	-	-	-	-	-	-
Missing	-	-	-	-	-	-	-	-

* Percentages are based on non-missing observations. Missing is reported as the percentage of the total group.

** Prostate cancer is based on D’Amico score, going 1, 2, 3, -1.

*** Score 5 for patients diagnosed with lung cancer is included.

### Initiating first-line cancer treatment

**Figure 2:**
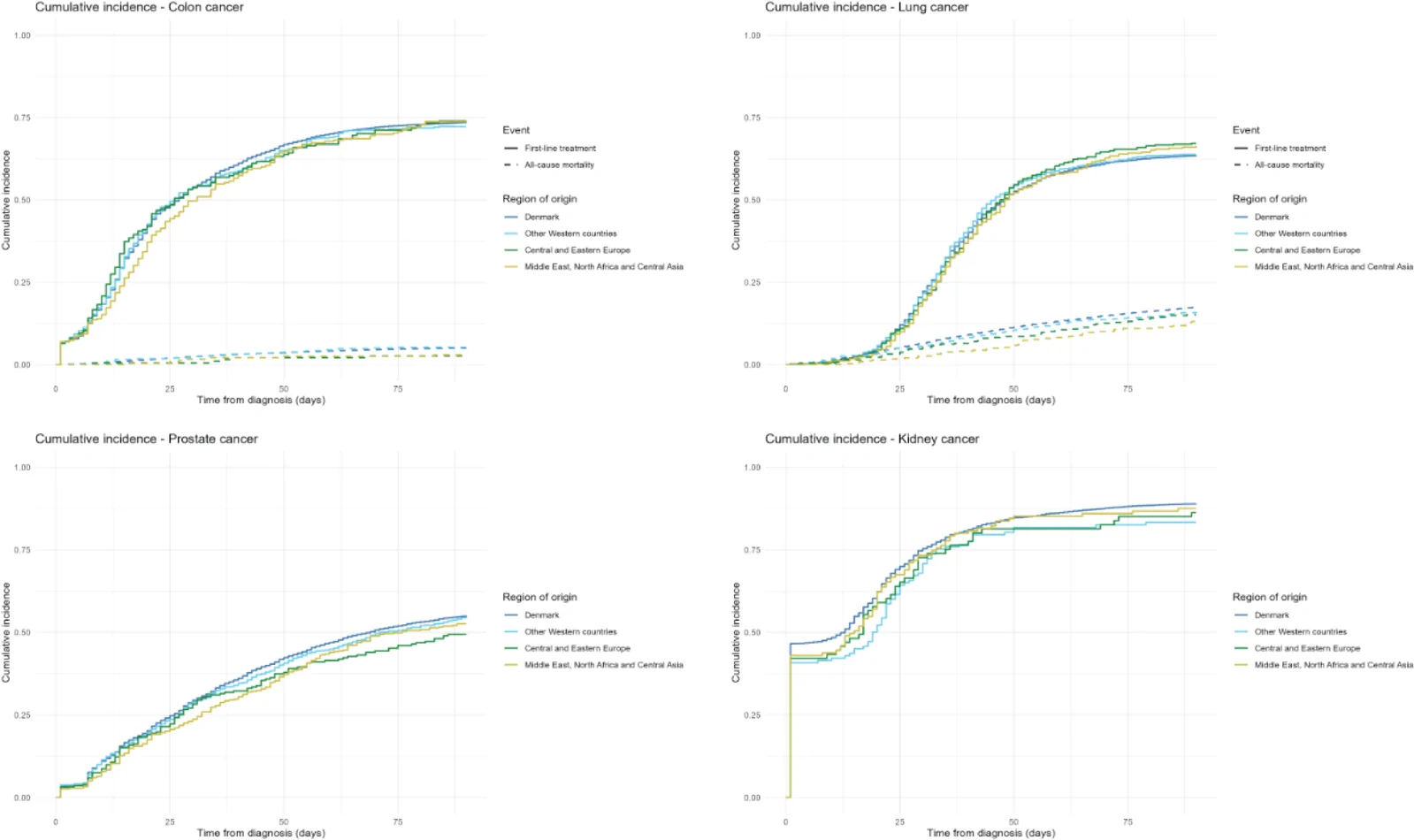
Cumulative incidence curves of first-line treatment within 90 days, with death as a competing event among patients diagnosed with colon, lung, prostate, and kidney cancer, by region of origin. The increase at time zero for kidney cancer reflects that diagnosis and first-line treatment are established on the same date. All-cause mortality is not shown for prostate and kidney cancer due to the few cases.

The unadjusted cumulative incidence curves of initiating first-line cancer treatment within 90 days (Figure 2) and the corresponding risk estimates at 30 and 90 days (Table 2) show no substantial differences between patients with an immigrant background and patients of Danish origin.

**Table 2:** Cumulative incidence of initiating first-line treatment at 30 and 90 days after diagnosis, stratified by cancer site.

Cancer site by region of origin	30-day			90-day		
	Risk % [95% CI]	Model 1 RR [95% CI]^a^	Model 2 RR [95% CI]^b^	Risk % [95% CI]	Model 1 RR [95% CI]^a^	Model 2 RR [95% CI]^b^
Colon cancer						
Denmark	54 [53; 55]	1.00 (ref.)	1.00 (ref.)	74 [73; 74]	1.00 (ref.)	1.00 (ref.)
Other Western countries	54 [49; 58]	1.01 [0.93; 1.10]	1.00 [0.95; 1.06]	72 [68; 76]	0.99 [0.94; 1.05]	0.99 [0.96; 1.02]
Central and Eastern Europe	54 [47; 61]	0.96 [0.85; 1.09]	1.00 [0.92; 1.08]	74 [68; 80]	0.97 [0.90; 1.05]	0.99 [0.93; 1.04]
Middle East, North Africa, and Central Asia	50 [43; 56]	0.87 [0.76; 0.99]	0.93 [0.85; 1.01]	74 [69; 80]	0.95 [0.88; 1.03]	0.98 [0.94; 1.03]
Lung cancer						
Denmark	22 [22; 23]	1.00 (ref.)	1.00 (ref.)	64 [63; 64]	1.00 (ref.)	1.00 (ref.)
Other Western countries	22 [19; 25]	0.96 [0.84; 1.10]	0.94 [0.82; 1.08]	64 [60; 67]	0.99 [0.94; 1.04]	0.97 [0.93; 1.02]
Central and Eastern Europe	20 [16; 24]	0.80 [0.66; 0.98]	0.76 [0.62; 0.93]	67 [63; 72]	0.96 [0.90; 1.02]	0.92 [0.87; 0.98]
Middle East, North Africa, and Central Asia	20 [15; 24]	0.81 [0.66; 1.01]	0.80 [0.64; 1.00]	66 [61; 71]	0.95 [0.89; 1.02]	0.97 [0.91; 1.02]
Prostate cancer						
Denmark	29 [29; 30]	1.00 (ref.)	1.00 (ref.)	55 [55; 56]	1.00 (ref.)	1.00 (ref.)
Other Western countries	29 [26; 32]	0.98 [0.88; 1.09]	0.99 [0.90; 1.10]	55 [51; 58]	1.00 [0.93; 1.06]	1.02 [0.96; 1.08]
Central and Eastern Europe	28 [23; 34]	0.98 [0.81; 1.19]	0.97 [0.80; 1.17]	49 [43; 56]	0.92 [0.81; 1.04]	0.94 [0.83; 1.06]
Middle East, North Africa, and Central Asia	24 [20; 28]	0.83 [0.70; 0.99]	0.87 [0.74; 1.02]	53 [48; 57]	0.98 [0.89; 1.07]	1.03 [0.94; 1.12]
Kidney cancer						
Denmark	75 [74; 76]	1.00 (ref.)	1.00 (ref.)	89 [88; 90]	1.00 (ref.)	1.00 (ref.)
Other Western countries	71 [63; 78]	0.95 [0.86; 1.05]	0.94 [0.85; 1.04]	83 [77; 90]	0.94 [0.88; 1.01]	0.93 [0.87; 1.00]
Central and Eastern Europe	73 [63; 82]	0.93 [0.81; 1.06]	0.94 [0.83; 1.06]	86 [79; 94]	0.94 [0.86; 1.02]	0.95 [0.87; 1.03]
Middle East, North Africa, and Central Asia	73 [66; 81]	0.94 [0.85; 1.04]	0.96 [0.87; 1.06]	88 [82; 93]	0.95 [0.90; 1.01]	0.98 [0.92; 1.04]

RR: risk ratio; CI: confidence interval.

a Adjusted for sex, age, and year of diagnosis.

b Additionally adjusted for cohabitation status, education, CCI, psychiatric disorders, stage, and cancer-specific variables.

The fully adjusted RRs of initiating first-line cancer treatment (model 2) suggest that patients from other Western countries than Denmark had a similar probability of initiating first-line treatment as patients of Danish origin (Table 2). Full adjustment (model 2) resulted only in minor changes to the age-, sex-, and period-adjusted analyses (model 1). For lung cancer, a 24% lower probability of first-line treatment was observed at 30 days (RR: 0.76 [95% CI: 0.62; 0.93]) for patients from Central and Eastern Europe compared with patients of Danish origin. This difference was smaller at 90 days but remained statistically significant (RR: 0.92 [95% CI: 0.87; 0.98]). For patients from the Middle East, North Africa, and Central Asia, adjusted analyses showed a lower probability of first-line lung cancer treatment at 30 days (RR: 0.80 [95% CI: 0.64; 1.00]), but not at 90 days.

When stratifying by stage at diagnosis (Tables S8–S11), the results were similar to the main analysis. However, the disparities remained statistically significant only among lung cancer patients from Central and Eastern Europe (RR_30 days_ 0.74 [95% CI: 0.59; 0.92] and RR_90 days_ 0.90 [95% CI: 0.83; 0.98]) and the Middle East, North Africa, and Central Asia (RR_30 days_ 0.75 [95% CI: 0.58; 0.97]) with advanced stage.

### All-cause mortality

Patients were followed for 4.4–5.0 median years across the four cancer sites, with the longest follow-up period among patients of Danish origin (Table S12). Overall, unadjusted mortality risks were comparable between patients of Danish origin and patients from other Western countries, whereas patients from Central and Eastern Europe and from the Middle East, North Africa, and Central Asia tended to have a slightly lower 5-year mortality (Table 3).

**Table 3:** Cumulative incidence of death at 1 and 5 years after diagnosis, stratified by cancer site.

Cancer site by region of origin	1 year			5 years		
	Risk % [95% CI]	Model 1 RR [95% CI]^a^	Model 2 RR [95% CI]^b^	Risk % [95% CI]	Model 1 RR [95% CI]^a^	Model 2 RR [95% CI]^b^
Colon cancer						
Denmark	17 [16; 17]	1.00 (ref.)	1.00 (ref.)	37 [36; 38]	1.00 (ref.)	1.00 (ref.)
Other Western countries	15 [11; 18]	0.85 [0.68; 1.06]	0.88 [0.75; 1.04]	35 [30; 40]	0.91 [0.80; 1.04]	0.93 [0.85; 1.01]
Central and Eastern Europe	15 [10; 20]	1.16 [0.84; 1.61]	0.97 [0.75; 1.25]	29 [22; 36]	0.98 [0.79; 1.21]	0.88 [0.74; 1.04]
Middle East, North Africa, and Central Asia	13 [8; 17]	1.02 [0.73; 1.42]	0.92 [0.69; 1.21]	28 [21; 35]	0.97 [0.78; 1.20]	0.95 [0.81; 1.12]
Lung cancer						
Denmark	49 [48; 49]	1.00 (ref.)	1.00 (ref.)	78 [78; 79]	1.00 (ref.)	1.00 (ref.)
Other Western countries	48 [45; 52]	1.00 [0.92; 1.07]	1.01 [0.96; 1.07]	73 [69; 77]	0.95 [0.91; 1.00]	0.98 [0.95; 1.01]
Central and Eastern Europe	39 [34; 44]	0.84 [0.74; 0.95]	0.85 [0.77; 0.93]	70 [64; 76]	0.95 [0.89; 1.02]	0.96 [0.92; 1.01]
Middle East, North Africa, and Central Asia	47 [41; 52]	0.98 [0.87; 1.10]	0.96 [0.88; 1.05]	80 [75; 85]	1.04 [0.98; 1.11]	1.02 [0.97; 1.07]
Prostate cancer						
Denmark	4 [4; 4]	1.00 (ref.)	1.00 (ref.)	22 [22; 23]	1.00 (ref.)	1.00 (ref.)
Other Western countries	3 [2; 5]	1.17 [0.79; 1.73]	0.99 [0.65; 1.50]	22 [19; 26]	1.13 [0.98; 1.30]	1.16 [1.01; 1.34]
Central and Eastern Europe	3 [1; 5]	0.88 [0.39; 1.98]	0.96 [0.42; 2.21]	16 [10; 22]	1.02 [0.74; 1.41]	1.02 [0.74; 1.42]
Middle East, North Africa, and Central Asia	2 [1; 4]	1.16 [0.58; 2.32]	1.23 [0.66; 2.32]	15 [10; 19]	0.92 [0.70; 1.19]	1.02 [0.80; 1.29]
Kidney cancer						
Denmark	11 [11; 12]	1.00 (ref.)	1.00 (ref.)	31 [29; 32]	1.00 (ref.)	1.00 (ref.)
Other Western countries	9 [4; 15]	0.84 [0.49; 1.43]	1.01 [0.64; 1.60]	27 [17; 36]	0.93 [0.67; 1.29]	1.04 [0.84; 1.29]
Central and Eastern Europe	6 [1; 12]	0.71 [0.30; 1.66]	0.76 [0.39; 1.49]	19 [8; 29]	0.72 [0.44; 1.17]	0.74 [0.51; 1.08]
Middle East, North Africa, and Central Asia	5 [1; 8]	0.44 [0.19; 1.00]	0.75 [0.39; 1.45]	20 [11; 28]	0.72 [0.46; 1.14]	0.91 [0.64; 1.29]

RR: risk ratio; CI: confidence interval.

a Adjusted for sex, age, and year of diagnosis.

b Additionally adjusted for cohabitation status, education, CCI, psychiatric disorders, stage, and cancer-specific variables.

In fully adjusted analyses (model 2), patients from other Western countries than Denmark diagnosed with prostate cancer had a higher risk of death 5 years after diagnosis (RR: 1.16 [95% CI: 1.01; 1.34]). The 1-year mortality was lower following lung cancer diagnosis (RR: 0.85 [95% CI: 0.77; 0.93]) among patients from Central and Eastern Europe compared with patients of Danish origin, but the 5-year mortality did not differ. No statistically significant differences were observed for patients from the Middle East, North Africa, and Central Asia (Table 3). Again, additional adjustment for sociodemographic and clinical characteristics resulted in only minor changes to the estimates.

For colon and lung cancer, when stratified by stage at diagnosis (Tables S13–14), similar results were observed. The lower mortality at 1 year after lung cancer diagnosis among patients from Central and Eastern Europe was found among both local/regional and advanced stage (RR: 0.61 [95% CI: 0.38; 1.00] and RR: 0.86 [95% CI: 0.78; 0.95]). Due to the few cases, the analysis was not performed for prostate and kidney cancer.

### Years in Denmark

Analyses for the probability of initiating first-line cancer treatment were performed with time in Denmark as the exposure (Table S15). Across all cancer sites, no statistically significant differences were found.

## Discussion

### Main findings

In this nationwide register-based cohort study, we found no systematic differences in initiation of guideline-recommended first-line cancer treatment or survival between patients with an immigrant background and patients of Danish origin. Although some statistically significant differences were observed, these were not consistent across cancer types or regions of origin. Adjustment for sociodemographic and clinical characteristics did not change these patterns.

### First-line cancer treatment

To our knowledge, this is the first Danish study to investigate differences in the initiation of first-line cancer treatment between patients with an immigrant background and patients of Danish origin. Furthermore, treatment initiation was assessed at predefined clinically relevant time points after diagnosis. Among patients with lung cancer, a difference in initiating first-line treatment was observed for patients from Central and Eastern Europe and the Middle East, North Africa, and Central Asia, particularly evident at 30 days post-diagnosis, with a 24 and 20% lower probability of initiating first-line cancer treatment, respectively. In line with this, a Norwegian study has reported delayed cancer treatment among non-Western immigrants diagnosed with lung cancer [35]. Despite these few trends, our results overall indicate that patients with an immigrant background in Denmark have the same probability of receiving first-line treatment as patients of Danish origin. This is consistent with a Danish study on acute myeloid leukemia, which found no differences in time to treatment or treatment allocation [15]. In contrast, American studies have consistently reported treatment disparities among racial and ethnic minorities [36], underscoring how inequalities vary across healthcare systems. In Denmark, cancer care is tax-financed and organized in standardized pathways [8], reducing the risk of suboptimal treatment due to financial constraints or limited access to healthcare services.

### All-cause mortality

Like previous Danish studies on mortality after a cancer diagnosis, which have primarily used broad categorizations of immigrant background, we found no overall systematic differences in mortality [14–17]. This contrasts with findings from American studies, where higher mortality has been documented among racial and ethnic minority groups [36], potentially reflecting differences in healthcare systems, access, and structural contexts. Consistent with findings from Nordic studies [37, 38], there was a tendency toward similar survival between patients of Danish origin and other Western-born patients and toward similar or better survival among non-Western-born patients, with a few exceptions. However, only two statistically significant associations were identified. Patients diagnosed with lung cancer from Central and Eastern Europe had a 15% lower risk of death 1 year after diagnosis, a difference no longer seen at 5 years. Within immigrant health research, the healthy immigrant effect – referring to the initially better health among immigrants – and salmon bias – where individuals with poor health return to their country of origin – are often used to explain observed survival advantages. However, the survival advantage typically diminishes with longer duration of residence [39]. In the present cohort, the median duration of residence in Denmark was 23–27 years, and emigration rates were low (≤2%), making these explanations less likely. The observed survival advantage is therefore not readily explained. Residual confounding or unmeasured factors may contribute, but the underlying mechanisms remain unclear. Unexpectedly, we observed a 16% increased risk of death 5 years after diagnosis for prostate cancer patients from other Western countries than Denmark. However, this finding was not consistent across cancer sites and may reflect a chance finding in the context of multiple comparisons.

### Strengths and limitations

A major strength of this study is the use of a nationwide cohort from the Danish clinical cancer databases, covering almost all patients diagnosed with the included cancer types over a long study period. The databases are required to cover at least 90% of eligible patients [40], and DCCG and DLCR report coverage exceeding 95% [24, 25]. Furthermore, the databases provide clinically validated and highly complete information on tumor stage, ECOG performance status, and treatment [24–27]. Linkage via the unique personal identification number (CPR) enabled inclusion of detailed sociodemographic and health-related data from high-quality national registers [28] for all residents in Denmark, irrespective of immigration background, allowing adjustment for important covariates. Standard first-line treatment was defined according to national guidelines, and regression models accounting for competing risks were applied [32]. Additionally, immigrants were categorized by regions of origin rather than the common Western versus non-Western dichotomy, allowing a more nuanced analysis.

Limitations include the lack of information on residence permit type, which may capture important differences in migration background. Although such data exist in the Danish registers, coverage is incomplete for immigrants arriving before 1997 [29], representing most of our cohort. Language barriers may also affect cancer care [41], but interpreter use is poorly captured in Danish registers. We therefore used the duration of residence in Denmark as a proxy for language barriers and awareness of available healthcare services. However, the small number of patients with shorter residence prevented a more detailed categorization by years since immigration. The relatively small immigrant population in Denmark also limited the number of patients from some regions of origin. Consequently, these regions (Southeast Asia and Pacific Western, Sub-Saharan Africa, and Latin America and the Caribbean [*N* = 608]) could not be analyzed, and our findings cannot be generalized to patients from the excluded regions. Furthermore, the recorded date of diagnosis may not reflect the actual clinical diagnosis. Thus, 30- and 90-day time points were used as pragmatic proxies for short and long time to treatment initiation, with treatment within 90 days considered clinically acceptable. For all analyses, the combination of small immigrant groups and multiple testing increases the uncertainty of the estimates and the risk of type I errors. Few death events among kidney and prostate cancer patients further contribute to this uncertainty. We therefore focused on overall patterns rather than isolated statistically significant findings.

### Clinical practice

As immigrant populations continue to grow and age globally [1, 23], more individuals with an immigrant background will require cancer care, making continued comparison of care and outcomes essential. With advancing age and increasing disease burden, structural, linguistic, and cultural barriers may have a greater impact on health outcomes and care experiences [23]. Although we found no clear systematic differences in first-line treatment or survival, previous studies have shown that immigrant patients may experience psychosocial disadvantages during cancer care, particularly related to communication and decision-making [42, 43], highlighting that equity cannot be captured by register data alone.

The immigrant population is dynamic, and future immigrants may differ from previous groups, and the generalizability of current findings should be interpreted with caution. Future research should extend analyses to other cancer sites, including those with higher prevalence among immigrant populations, such as liver and stomach cancer [23], which might be preventable.

## Conclusion

In this study, we found no systematic differences in first-line cancer treatment initiation or survival between patients with an immigrant background and patients of Danish origin. While we found some statistically significant differences, we found no consistent pattern across cancer types or regions of origin. This finding might be a positive indication of equitable access to cancer care. However, other important aspects of cancer care, such as shared decision-making, patient preferences, and communication barriers, need further investigation.

## Supplementary Material



## Data Availability

The data utilized in this study were accessed remotely on a secure platform at Statistics Denmark. The data that support the findings of this study are available for collaborative research projects upon reasonable request.
