# Supplementary material for: Disparities in initiating standard first-line cancer treatment and survival for patients with an immigrant background: a Danish register-based cohort study
Source: Acta Oncol. 2026 Sep 18;65:46256. doi: 10.2340/1651-226X.2026.46256 (PMC13595832; doi:10.2340/1651-226X.2026.46256)
Supplement: Supplementary file 1 [file AO-65-46256-s1.pdf]

Supplementary material has been published as submitted. It has not been copyedited, or typeset by Acta Oncologica

Supplementary material

Table of contents

Table S1: Categorization of region of origin 2

Table S2: First-line treatment of non-small cell lung cancer 3

Table S3: First-line treatment of colon cancer 3

Table S4: First-line treatment of kidney cancer 4

Table S5: First-line treatment of prostate cancer 4

Table S6: Categorization of local/regional and advanced stage 4

Table S7: Most common countries 5

Table S8: Cumulative incidence of initiating first-line treatment at 30 and 90 days after diagnosis of colon cancer, stratified by stage 5

Table S9: Cumulative incidence of initiating first-line treatment at 30 and 90 days after diagnosis of lung cancer, stratified by stage 6

Table S10: Cumulative incidence of initiating first-line treatment at 30 and 90 days after diagnosis of prostate cancer, stratified by stage 6

Table S11: Cumulative incidence of initiating first-line treatment at 30 and 90 days after diagnosis of kidney cancer, stratified by stage 7

Table S12: Median (IQR) follow-up time for each cancer site 7

Table S13: Cumulative incidence of death at 1 and 5 years after diagnosis of colon cancer, stratified by stage 7

Table S14: Cumulative incidence of death at 1 and 5 years after diagnosis of lung cancer, stratified by stage 8

Table S15: Cumulative incidence of initiating first-line treatment at 30 and 90 days after diagnosis based on years since immigration 8

Table S1: Categorization of region of origin

**Region of origin**	**Included countries**	**Final categorization of region of origin**
Denmark	Denmark, Greenland, Faroe Islands	Denmark
Middle East and North Africa	Algeria, Bahrain, Egypt, Iran, Iraq, Israel, Jordan, Kuwait, Lebanon, Libya, Mauritania, Morocco, Oman, Palestine (including Gaza, West Bank, East Jerusalem), Qatar, Saudi Arabia, Syria, Tunisia, Turkey, United Arab Emirates, Yemen	Middle East, North Africa and Central Asia
Central and Eastern Europe	Albania, Armenia, Belarus, Bosnia and Herzegovina, Bulgaria, Croatia, Czechoslovakia, Czech Republic, Estonia, Georgia, Hungary, Kosovo, Latvia Lithuania, Moldova, Montenegro, North Macedonia, Poland, Romania, Russian Federation, Serbia, Slovak Republic, Slovenia, Soviet Union, Ukraine, Yugoslavia	Central and Eastern Europe
Western (excluding Denmark)	Austria, Andorra, Belgium, Canada, Cyprus, Finland, France, Germany, Greece, Iceland, Ireland, Italy, Liechtenstein, Luxembourg, Malta, Monaco, Netherlands, Norway, Portugal, San Marino, Spain, Sweden, Switzerland, United Kingdom, Vatican state, The United States, Australia, New Zealand	Other Western Countries
Asia	Bangladesh, Bhutan, Brunei, Cambodia, Cook Islands, Darussalam, Fiji, Indonesia, Islands of the Pacific Ocean, Japan, Kiribati, Korea Dem. People's Rep., Korea Rep., Laos, Malaysia, Maldives, Marshall Islands, Myanmar, Nauru, Nepal, Papua New Guinea, Philippines, Samoa, Singapore, Solomon Islands, Sri Lanka, Taiwan, Thailand, Timor-Leste, Tonga, Tuvalu, Vanuatu, Vietnam, China, India	Other area (not included)
Central Asia	Afghanistan, Azerbaijan, Kazakhstan, Kyrgyz Republic, Mongolia, Pakistan, Tajikistan, Turkmenistan, Uzbekistan	Middle East, North Africa and Central Asia
Sub-Saharan Africa	Angola, Benin, Botswana, Burkina Faso, Burundi, Cabo Verde, Cameroon, Central African Republic, Chad, Comoros, Congo Dem. Rep., Congo Rep., Cote d'Ivoire, Djibouti, Equatorial Guinea, Eritrea, Eswatini, Ethiopia, Gabon, Gambia, Ghana, Guinea, Guinea-Bissau, Kenya, Lesotho, Liberia, Madagascar, Malawi, Mali, Mauritius, Mozambique, Namibia, Niger, Nigeria, Rwanda, São Tomé and Príncipe, Senegal, Seychelles, Sierra Leone, Somalia, South Africa, South Sudan, Sudan, Tanzania, Togo, Uganda, Zambia, Zimbabwe	Other area (not included)
Latin America and the Caribbean	Antigua and Barbuda, Argentina, Bahamas, Barbados, Belize, Bolivia, Brazil, Chile, Colombia, Costa Rica, Cuba, Dominica, Dominican Republic, Ecuador, El Salvador, Grenada, Guatemala, Guyana, Haiti, Honduras, Jamaica, Mexico, Nicaragua, Panama, Paraguay, Peru, Saint Kitts and Nevis, Saint Lucia, Saint Vincent and the Grenadines, Suriname, The United States Virgin Islands, Trinidad and Tobago, Uruguay, Venezuela	Other area (not included)

Table S2: First-line treatment of non-small cell lung cancer

TNM stage	Stage	Differences	First-line treatment	Time
T1-2N0M0	I-IIA	Performance status 0-2	Surgery / Stereotactic radiation / radiotherapy + chemotherapy / Ablation	Treatment given within 3 months after diagnosis
T3-4N0M0	IIB-IIIA	Performance status 0-1	Surgery / Chemotherapy / Immunotherapy / Radiotherapy + chemotherapy	Treatment given within 3 months after diagnosis
T3-4N0M0	IIB-IIIA	Performance status 2	Surgery / Chemotherapy / Radiotherapy + chemotherapy	Treatment given within 3 months after diagnosis
T0-3N1M0	IIB-IIIA	Performance status 0-1	Surgery / Chemotherapy / Immunotherapy / Radiotherapy + chemotherapy	Treatment given within 3 months after diagnosis
T0-3N1M0	IIB-IIIA	Performance status 2	Radiotherapy + chemotherapy / Chemotherapy	Treatment given within 3 months after diagnosis
T0-3N2-3M0	IIIA-IIIC	Performance status 0-2[a]	Radiotherapy + chemotherapy / Chemotherapy / Immunotherapy	For stage IIIA, Treatment given within 3 months after diagnosis, for stage IIIB-IIIC given within 6 months after diagnosis
T4N1-3M0	IIIA-IIIC	Performance status 0-2[a]	Radiotherapy + chemotherapy / Chemotherapy / Immunotherapy	For stage IIIA, Treatment given within 3 months after diagnosis, for stage IIIB-IIIC given within 6 months after diagnosis
TanyNanyM1	IVA-IVB	Performance status 0-2[a]	Radiotherapy + chemotherapy / Chemotherapy / Immunotherapy / Surgery	Treatment given within 6 months after diagnosis

[a] Except no immunotherapy is given to patients with performance status 2.

Table S3: First-line treatment of colon cancer

TNM stage	Stage	Differences*	First-line treatment	Time
T1-2N0M0	I		Surgery	<90 days after diagnosis
T3-4N0M0	II	Not for those with dMMR or non-operable by surgeons' evaluation with the patient[a]. Additional risk factors: leakage[b], <12 lymph nodes[b], T4, and signet ring cell carcinoma.	Surgery / Adjuvant chemotherapy (combination 5FU /Oxali)	Surgery <90 days after diagnosis, adjuvant chemotherapy < 3 months after operation
TanyN1-2+M0	III	Not for those with dMMR or non-operable by surgeons' evaluation with the patient[a].	Surgery + Adjuvant chemotherapy (combination 5FU /Oxali)	Surgery <90 days after diagnosis, adjuvant chemotherapy < 3 months after operation
TanyNany-1M1	IV		Surgery / Chemotherapy / Radiotherapy / Radiotherapy + chemotherapy	Any treatment initiated <90 days after diagnosis.

* Age and performance status also relevant for treatment decisions
[a] Not available in the registers
[b] Not included due to the timing of measurement causing immortal time bias

Table S4: First-line treatment of kidney cancer

TNM stage	Differences	First-line treatment	Time
T1N0M0	Age, comorbidities	Partial nephrectomy / Ablation / Active surveillance / Nephrectomy	Any treatment initiated <90 days after diagnosis.
T2-3N0M0	Age, comorbidities	Nephrectomy / Partial nephrectomy	Any treatment initiated <90 days after diagnosis.
T4N0M0	Age, comorbidities	Nephrectomy / Immunotherapy/ Watchful waiting	Any treatment initiated <90 days after diagnosis.
TanyN1M0	Age, comorbidities	Nephrectomy / Immunotherapy / Watchful waiting / Tyrosine kinase inhibitors	Any treatment initiated <90 days after diagnosis.
TanyNanyM1	Age, comorbidities	Nephrectomy / Immunotherapy / Radiotherapy / Watchful waiting / Tyrosine kinase inhibitors	Any treatment initiated <90 days after diagnosis.

Table S5: First-line treatment of prostate cancer

TNM stage	D'Amico	Differences	First-line treatment	Time
T1c-T2a, PSA≤10, Gleason score≤6	Low	Age (+/- 70 years) and overall health (comorbidities)	Active surveillance / Surgery / Brachytherapy / Radiotherapy	Any treatment initiated <90 days after diagnosis.
T2b, 10< PSA≤20, Gleason score 7	Intermediary	Age (+/- 70 years) and overall health (comorbidities)	Active surveillance / Surgery / Brachytherapy / Radiotherapy	Any treatment initiated <90 days after diagnosis.
≥T2c, PSA>20, Gleason score >7	High	Age (+/- 70 years) and overall health (comorbidities). If heart disease, no hormone therapy	Radiotherapy / Chemotherapy / Hormone therapy / Watchful waiting	Any treatment initiated <90 days after diagnosis.
T3-4 / N1 / M1	(Score of -1)	Age (+/- 70 years) and overall health (comorbidities). If heart disease, no hormone therapy	Radiotherapy / Chemotherapy / Hormone therapy / Watchful waiting	Any treatment initiated <90 days after diagnosis.

Table S6: Categorization of local/regional and advanced stage

<table>
<tr><th>Cancer Stage</th><th>Colorectal</th><th colspan="2">Lung</th><th>Prostate</th><th>Kidney</th></tr>
<tr><td>I</td><td>Local/regional stage</td><td colspan="2">Local/regional stage</td><td>Local/regional stage (D'Amico low)</td><td>Local/regional stage</td></tr>
<tr><td>II</td><td>Local/regional stage</td><td colspan="2">Local/regional stage</td><td>Local/regional stage (D'Amico intermedia)</td><td>Local/regional stage</td></tr>
<tr><td rowspan="2">III</td><td rowspan="2">Local/regional stage</td><td>IIIA</td><td>Local/regional stage</td><td rowspan="2">Advanced stage (D'Amico high)</td><td rowspan="2">Local/regional stage</td></tr>
<tr><td>IIIB-C</td><td>Advanced stage</td></tr>
<tr><td>IV</td><td>Advanced stage</td><td colspan="2">Advanced stage</td><td>Advanced stage (D'Amico out of categorization)</td><td>Advanced stage</td></tr>
</table>

Table S7: Most common countries

Placement	Colon cancer	n	Lung cancer	n	Prostate cancer	n	Kidney cancer	n
First	Germany	152	Germany	262	Germany	234	Turkey	40
Second	Norway	94	Bosnia and Herzegovina	127	United Kingdom	136	Germany	31
Third	Turkey	76	Norway	115	Sweden	101	Norway	26
Forth	Sweden	63	Turkey	110	Turkey	96	Bosnia and Herzegovina	24
Fifth	United Kingdom	58	Sweden	99	Poland	78	Sweden	22
Sixth	Bosnia and Herzegovina	51	Poland	98	Norway	77	Iraq	20
Seventh	Poland	48	United Kingdom	79	Iran	64	Poland	20
Eight	Yugoslavia	36	Yugoslavia	65	Bosnia and Herzegovina	60	Iran	15
Ninth	Iraq	34	Lebanon	53	Iraq	51	Pakistan	15
Tenth	Iran	34	Iraq	38	The United States	50	United Kingdom	14

Table S8: Cumulative incidence of initiating first-line treatment at 30 and 90 days after diagnosis of colon cancer, stratified by stage

	RR 30 days, Model 1 [95%CI]	RR 30 days, Model 2 [95%CI]	RR 90 days, Model 1 [95%CI]	RR 90 days, Model 2 [95%CI]
Local/regional stage				
Denmark	1.00 (ref.)	1.00 (ref.)	1.00 (ref.)	1.00 (ref.)
Other Western countries	1.01 [0.92;1.11]	1.01 [0.96;1.07]	1.00 [0.95;1.06]	1.00 [0.97;1.03]
Central and Eastern Europe	0.92 [0.79;1.08]	0.98 [0.90;1.07]	0.96 [0.88;1.05]	0.97 [0.92;1.03]
Middle East, North Africa and Central Asia	0.86 [0.74;1.00]	0.92 [0.84;1.01]	0.95 [0.87;1.03]	0.98 [0.94;1.02]
Advanced stage				
Denmark	1.00 (ref.)	1.00 (ref.)	1.00 (ref.)	1.00 (ref.)
Other Western countries	1.00 [0.84;1.19]	0.99 [0.84;1.17]	0.98 [0.87;1.10]	0.97 [0.87;1.07]
Central and Eastern Europe	1.07 [0.86;1.33]	1.02 [0.82;1.28]	1.02 [0.88;1.18]	0.99 [0.86;1.13]
Middle East, North Africa and Central Asia	0.93 [0.71;1.22]	0.98 [0.76;1.26]	0.98 [0.84;1.15]	1.02 [0.88;1.18]

Table S9: Cumulative incidence of initiating first-line treatment at 30 and 90 days after diagnosis of lung cancer, stratified by stage

	RR 30 days, Model 1 [95%CI]	RR 30 days, Model 2 [95%CI]	RR 90 days, Model 1 [95%CI]	RR 90 days, Model 2 [95%CI]
Local/regional stage				
Denmark	1.00 (ref.)	1.00 (ref.)	1.00 (ref.)	1.00 (ref.)
Other Western countries	0.97 [0.75;1.25]	0.98 [0.76;1.26]	0.95 [0.89;1.01]	0.95 [0.90;1.01]
Central and Eastern Europe	0.69 [0.45;1.06]	0.68 [0.44;1.05]	0.96 [0.88;1.04]	0.95 [0.88;1.02]
Middle East, North Africa and Central Asia	0.96 [0.66;1.40]	0.94 [0.64;1.38]	1.01 [0.94;1.09]	1.01 [0.94;1.08]
Advanced stage				
Denmark	1.00 (ref.)	1.00 (ref.)	1.00 (ref.)	1.00 (ref.)
Other Western countries	0.96 [0.82;1.13]	0.91 [0.78;1.07]	1.03 [0.95;1.11]	1.00 [0.95;1.07]
Central and Eastern Europe	0.82 [0.66;1.02]	0.74 [0.59;0.92]	0.98 [0.89;1.07]	0.90 [0.83;0.98]
Middle East, North Africa and Central Asia	0.75 [0.58;0.97]	0.75 [0.58;0.97]	0.91 [0.82;1.02]	0.94 [0.86;1.03]

Table S10: Cumulative incidence of initiating first-line treatment at 30 and 90 days after diagnosis of prostate cancer, stratified by stage

	RR 30 days, Model 1 [95%CI]	RR 30 days, Model 2 [95%CI]	RR 90 days, Model 1 [95%CI]	RR 90 days, Model 2 [95%CI]
Local/regional stage				
Denmark	1.00 (ref.)	1.00 (ref.)	1.00 (ref.)	1.00 (ref.)
Other Western countries	1.15 [0.92;1.45]	1.13 [0.89;1.43]	1.02 [0.92;1.14]	1.02 [0.92;1.13]
Central and Eastern Europe	1.26 [0.89;1.79]	1.34 [0.95;1.91]	0.84 [0.69;1.03]	0.86 [0.70;1.05]
Middle East, North Africa and Central Asia	0.83 [0.60;1.15]	0.87 [0.62;1.22]	0.87 [0.75;1.00]	0.91 [0.79;1.04]
Advanced stage				
Denmark	1.00 (ref.)	1.00 (ref.)	1.00 (ref.)	1.00 (ref.)
Other Western countries	0.97 [0.86;1.08]	0.98 [0.88;1.10]	0.99 [0.92;1.07]	1.01 [0.94;1.08]
Central and Eastern Europe	0.94 [0.76;1.17]	0.95 [0.76;1.19]	0.91 [0.78;1.06]	0.94 [0.81;1.10]
Middle East, North Africa and Central Asia	0.89 [0.74;1.07]	0.92 [0.76;1.11]	0.99 [0.88;1.11]	1.03 [0.92;1.14]

Table S11: Cumulative incidence of initiating first-line treatment at 30 and 90 days after diagnosis of kidney cancer, stratified by stage

	RR 30 days, Model 1 [95%CI]	RR 30 days, Model 2 [95%CI]	RR 90 days, Model 1 [95%CI]	RR 90 days, Model 2 [95%CI]
Local/regional stage				
Denmark	1.00 (ref.)	1.00 (ref.)	1.00 (ref.)	1.00 (ref.)
Other Western countries	0.97 [0.88;1.08]	0.96 [0.87;1.06]	0.94 [0.87;1.02]	0.94 [0.87;1.01]
Central and Eastern Europe	0.96 [0.84;1.10]	0.97 [0.86;1.10]	0.99 [0.92;1.06]	1.00 [0.93;1.08]
Middle East, North Africa and Central Asia	0.92 [0.83;1.03]	0.95 [0.86;1.06]	0.94 [0.88;1.00]	0.97 [0.90;1.03]
Advanced stage				
Denmark	1.00 (ref.)	1.00 (ref.)	1.00 (ref.)	1.00 (ref.)
Other Western countries	0.82 [0.57;1.17]	0.81 [0.57;1.16]	0.94 [0.75;1.17]	0.93 [0.74;1.16]
Central and Eastern Europe	0.80 [0.55;1.18]	0.81 [0.55;1.19]	0.74 [0.53;1.03]	0.74 [0.54;1.03]
Middle East, North Africa and Central Asia	1.07 [0.87;1.32]	1.05 [0.84;1.31]	1.06 [0.98;1.16]	1.06 [0.98;1.14]

Table S12: Median (IQR) follow-up time for each cancer site

	Patients diagnosed with colon cancer	Patients diagnosed with lung cancer	Patients diagnosed with prostate cancer	Patients diagnosed with kidney cancer
Overall	5 (2.5-5)	4.4 (2.1-5)	4.9 (2.3-5)	4.5 (2.1-5)
Denmark	5 (2.5-5)	4.4 (2.1-5)	4.9 (2.3-5)	4.6 (2.1-5)
Other Western Countries	5 (2.5-5)	4.3 (2.1-5)	4.5 (2.0-5)	3.8 (1.7-5)
Central and Eastern Europe	4.3 (1.8-5)	3.5 (1.3-5)	4.6 (2.0-5)	3.8 (1.5-5)
Middle East, North Africa and Central Asia	4.1 (1.8-5)	4.2 (1.9-5)	3.9 (1.7-5)	4.4 (2.0-5)

Table S13: Cumulative incidence of death at 1 and 5 years after diagnosis of colon cancer, stratified by stage

	RR 1 year, Model 1 [95%CI]	RR 1 year, Model 2 [95%CI]	RR 5 years, Model 1 [95%CI]	RR 5 years, Model 2 [95%CI]
Local/regional stage				
Denmark	1.00 (ref.)	1.00 (ref.)	1.00 (ref.)	1.00 (ref.)
Other Western countries	0.68 [0.40;1.15]	0.70 [0.42;1.18]	0.81 [0.63;1.04]	0.86 [0.68;1.09]
Central and Eastern Europe	1.56 [0.83;2.95]	1.42 [0.86;2.33]	0.99 [0.68;1.44]	0.84 [0.63;1.12]
Middle East, North Africa and Central Asia	1.41 [0.76;2.60]	1.31 [0.82;2.09]	1.03 [0.72;1.48]	0.88 [0.65;1.20]
Advanced stage				
Denmark	1.00 (ref.)	1.00 (ref.)	1.00 (ref.)	1.00 (ref.)
Other Western countries	0.91 [0.75;1.11]	0.91 [0.77;1.09]	0.97 [0.89;1.06]	0.95 [0.87;1.04]
Central and Eastern Europe	0.91 [0.67;1.23]	0.93 [0.70;1.22]	0.94 [0.80;1.11]	0.95 [0.81;1.12]
Middle East, North Africa and Central Asia	0.91 [0.64;1.29]	0.88 [0.63;1.22]	0.99 [0.82;1.18]	0.96 [0.81;1.14]

Table S14: Cumulative incidence of death at 1 and 5 years after diagnosis of lung cancer, stratified by stage

	RR 1 year, Model 1 [95%CI]	RR 1 year, Model 2 [95%CI]	RR 5 years, Model 1 [95%CI]	RR 5 years, Model 2 [95%CI]
Local/regional stage				
Denmark	1.00 (ref.)	1.00 (ref.)	1.00 (ref.)	1.00 (ref.)
Other Western countries	0.89 [0.68;1.18]	0.89 [0.69;1.16]	0.87 [0.75;1.00]	0.91 [0.80;1.04]
Central and Eastern Europe	0.59 [0.36;0.98]	0.61 [0.38;1.00]	0.85 [0.68;1.05]	0.87 [0.72;1.05]
Middle East, North Africa and Central Asia	0.93 [0.61;1.42]	0.91 [0.61;1.36]	1.11 [0.92;1.33]	1.15 [0.97;1.36]
Advanced stage				
Denmark	1.00 (ref.)	1.00 (ref.)	1.00 (ref.)	1.00 (ref.)
Other Western countries	1.01 [0.95;1.08]	1.02 [0.97;1.08]	0.99 [0.97;1.02]	1.00 [0.97;1.03]
Central and Eastern Europe	0.83 [0.74;0.92]	0.86 [0.78;0.95]	0.97 [0.93;1.02]	1.00 [0.95;1.04]
Middle East, North Africa and Central Asia	0.96 [0.87;1.07]	0.96 [0.88;1.05]	1.02 [0.97;1.06]	1.01 [0.97;1.05]

Table S15: Cumulative incidence of initiating first-line treatment at 30 and 90 days after diagnosis based on years since immigration

	RR 30 days, Model 1 [95%CI]	RR 30 days, Model 2 [95%CI]	RR 90 days, Model 1 [95%CI]	RR 90 days, Model 2 [95%CI]
Patients diagnosed with colon cancer				
Been in Denmark for >20 years	1.00 (ref.)	1.00 (ref.)	1.00 (ref.)	1.00 (ref.)
Been in Denmark for ≤20 years	0.99 [0.85;1.17]	1.01 [0.92;1.12]	1.01 [0.92;1.11]	1.00 [0.94;1.07]
Patients diagnosed with lung cancer				
Been in Denmark for >20 years	1.00 (ref.)	1.00 (ref.)	1.00 (ref.)	1.00 (ref.)
Been in Denmark for ≤20 years	1.19 [0.95;1.49]	1.24 [0.99;1.56]	0.98 [0.90;1.06]	0.99 [0.92;1.07]
Patients diagnosed with prostate cancer				
Been in Denmark for >20 years	1.00 (ref.)	1.00 (ref.)	1.00 (ref.)	1.00 (ref.)
Been in Denmark for ≤20 years	0.89 [0.70;1.12]	0.84 [0.66;1.05]	1.05 [0.93;1.20]	1.01 [0.89;1.14]
Patients diagnosed with kidney cancer				
Been in Denmark for >20 years	1.00 (ref.)	1.00 (ref.)	1.00 (ref.)	1.00 (ref.)
Been in Denmark for ≤20 years	1.02 [0.88;1.17]	1.05 [0.92;1.19]	1.01 [0.93;1.11]	1.04 [0.96;1.13]
